# 3,5-Dicaffeoylquinic Acid Lowers 3T3-L1 Mitotic Clonal Expansion and Adipocyte Differentiation by Enhancing Heme Oxygenase-1 Expression

**DOI:** 10.3390/molecules26165027

**Published:** 2021-08-19

**Authors:** Alice Raineri, Rachele Campagnari, Roberto Dal Toso, Stefano Copetti, Macarena Gomez-Lira, Marta Menegazzi

**Affiliations:** 1 Department of Neurosciences, Biomedicine and Movement Sciences, School of Medicine, University of Verona, Strada Le Grazie, 8, 37134 Verona, Italy; alice.raineri@univr.it (A.R.); rachele.campagnari@univr.it (R.C.); macarena.gomezlira@univr.it (M.G.-L.); 2 Croda Italiana S.p.A., Via Pietro Grocco, 27036 Mortara, Italy; roberto.daltoso.rdt@gmail.com (R.D.T.); stefano.copetti@croda.com (S.C.)

**Keywords:** adipogenesis, natural products, HO-1, Nrf2, AMPK, Akt, mitotic clonal expansion

## Abstract

Adipogenesis is a complex process in which cell commitment and mitotic clonal expansion (MCE) are in-sequence crucial events leading to terminal adipocyte differentiation. The molecules able to block some key signals in this cascade can hamper adipogenesis becoming promising agents to counteract hyperplasia and hypertrophy of adipose tissue. Mono- and di-caffeoylquinic acid isomers are biologically active polyphenols, displaying in vitro and in vivo antioxidant, hepatoprotective, anti-diabetic and anti-obesity properties. Among these isomers, 3,5-dicaffeoylquinic acid (DCQA) has been reported to inhibit lipid accumulation in adipose cells more successfully than others. Thus, we investigated DCQA effects and molecular mechanisms on 3T3-L1 pre-adipocytes induced to differentiate with a hormonal cocktail (MDI). Oil Red O incorporation assessed that DCQA pre-treatment inhibited lipid accumulation in 3T3-L1 cells induced to differentiate for 10 days. At this time, an increased phosphorylation of both AMP-activated kinase and acetyl-CoA carboxylase, as well as a strong decrease in fatty acid synthase protein level, were registered by immunoblotting, thereby suggesting that DCQA treatment can reduce fatty acid anabolism in 3T3-L1 adipocytes. Furthermore, BrdU incorporation assay, performed 48 h after hormonal stimulation, revealed that DCQA treatment was also able to hinder the 3T3-L1 cell proliferation during the MCE, which is an essential step in the adipogenic process. Thus, we focused our attention on early signals triggered by the differentiation stimuli. In the first hours after hormonal cocktail administration, the activation of ERK1/2 and Akt kinases, or CREB and STAT3 transcription factors, was not affected by DCQA pre-treatment. Whereas 24 h after MDI induction, DCQA pre-treated cells showed increased level of the transcription factor Nrf2, that induced the expression of the antioxidant enzyme heme oxygenase 1 (HO-1). In control samples, the expression level of HO-1 was reduced 24 h after MDI induction in comparison with the higher amount of HO-1 protein found at 2 h. The HO-1 decrease was functional by allowing reactive oxygen species to boost and allowing cell proliferation induction at the beginning of MCE phase. Instead, in DCQA-treated cells the HO-1 expression was maintained at high levels for a further 24 h; in fact, its expression decreased only 48 h after MDI stimulation. The longer period in which HO-1 expression remained high led to a delay of the MCE phase, with a subsequent inhibition of both C/EBP-α expression and adipocyte terminal differentiation. In conclusion, DCQA counteracting an excessive adipose tissue expansion may become an attractive option in obesity treatment.

## 1. Introduction

In light of its competence in secreting hormones and cytokines, adipose tissue has been recognized as the largest endocrine organ, which can balance cellular anabolic and catabolic metabolism, a key requirement to preserve energy homeostasis and total body health [1]. Conversely, adipose tissue dysfunction can arise in response to overall hyper-anabolic conditions, as occurs during obesity and metabolic syndrome [1].

Adipogenesis has been deeply investigated in vitro, especially thanks to 3T3-L1 pre-adipocyte cell culture by which the cascade of events eliciting the differentiation process can be followed. After stimulation with adipogenic hormonal cocktail (MDI), 3T3-L1 pre-adipocytes become phenotypically committed and enter in the mitotic clonal expansion (MCE) phase. Afterward, 3T3-L1 cells stop proliferation and cellular maturation as terminally differentiated adipocytes can occur [2]. Tang et al. showed that MCE is an essential event for 3T3-L1 differentiation into adipocytes [3]. In actuality, during such an event, post-confluent 3T3-L1 pre-adipocytes are driven to re-enter in the cell cycle by MDI, which elicits many proliferative signaling pathways [4]. Among others, the activation of extracellular signal-regulated kinases 1/2 (ERK1/2), protein kinase B (Akt), the signal transducer and activator of transcription (STAT)-3, and cyclic-AMP response element-binding protein (CREB), play a pivotal role [5,6]. Starting from these early steps, adipogenesis makes progress through a cascade of expression and activation of transcription factors such as the CCAAT/enhancer-binding protein (C/EBP)-β, C/EBP-δ, C/EBP-α and peroxisome proliferator-activated receptor (PPAR)-γ, leading to a final adipocyte maturation [7,8].

Lee et al. attested that reactive oxygen species (ROS), normally produced by adipocytes after MDI induction, facilitate cell differentiation by favoring the DNA-binding activity of C/EBP-β and accelerating the cell entry in S phase [9]. Antioxidant administration, instead, caused cell cycle arrest during the MCE [9]. This suggests that a significant oxidative stress condition is essential for adipogenesis.

Nuclear factor erythroid 2-related factor 2 (Nrf2) is a redox-sensitive transcription factor able to trigger a generalized cytoprotective response by binding to the antioxidant response element of many gene promoters [10]. In this way, Nrf2 elicits the transcription of a cluster of genes encoding for antioxidant enzymes [10]. Among these, heme oxygenase 1 (HO-1), which catalyzes heme degradation, has been reported to be a negative regulator of adipogenesis in 3T3-L1 cells [11]. Importantly, experiments using HO-1 knockout mice suggest a direct role of HO-1 in the commitment phase of adipose precursor cells [12]. Likewise, Br-deoxy-uridine (BrdU)-uptake assays in 3T3-L1 cells, engineered for HO-1 gain- and loss of function, further support an inhibitory role of HO-1 on the MDI-elicited MCE [12].

Another enzyme that influences adipocyte differentiation, though at a more advanced stage, is the AMP-activated protein kinase (AMPK). When AMPK is triggered, an inhibition of adipocyte differentiation occurs by switching off the C/EBP-α and PPAR-γ pro-adipogenic effects [4]. In actuality, AMPK activation inhibits the expression of fatty acid synthase (FAS) [13]. In addition, AMPK modulates enzyme activity or expression of acetyl-CoA carboxylase (ACC) and malonil CoA-decarboxilase. Thereby, AMPK activation can lower the cytosolic level of malonyl-CoA, which governs the equilibrium between fatty acids anabolism and catabolism [13]. Ultimately, AMPK activation reduces the lipid droplet amount stored in mature adipocytes, thus impairing a full energy accumulation as triglycerides.

Many researchers are looking for naturally occurring electrophilic compounds which are able to inhibit adipocyte differentiation or revert adipose distress [14]. Indeed, for many plant extracts and their secondary metabolites, a positive correlation between the antioxidant properties of these compounds and their anti-adipogenic effects is widely known. Both coffee and cocoa extracts reduced the production of ROS as well as the lipid accumulation in adipocytes [15]. *Hypericum perforatum* extract protects against diabetes complications through the inhibition of oxidative stress signaling [16], and it counteracts lipid accumulation by downregulating the expression of FAS and lipoprotein lipase [17].

Many studies report that natural products, such as curcumin, piperine, epigallocatechin gallate, berberine, quercetin and resveratrol, have been able to affect adipogenesis (reviewed by [18,19,20]) by activating AMPK [21,22,23], or by hindering STAT3- [5,23] and CREB [6,24,25]-activation pathways. Other works showed that an increase in Nrf2/HO-1 expression elicited by natural products could be a major determinant of their anti-adipogenic properties [26,27,28].

It should be mentioned that fractions from many plant sources rich in caffeoylquinic acid isomers seem to be highly active in the protection toward multi-organ dysfunction [29]. These biologically active dietary polyphenols actually show potential effects against inflammatory and oxidative-induced damage [30,31], type 1 and 2 diabetes complications [32,33], non-alcoholic fatty liver disease [34], hyperlipidemia in hamsters fed with a high-fat diet [35] and lipid accumulation in adipocytes [36,37,38].

**Figure 1:**
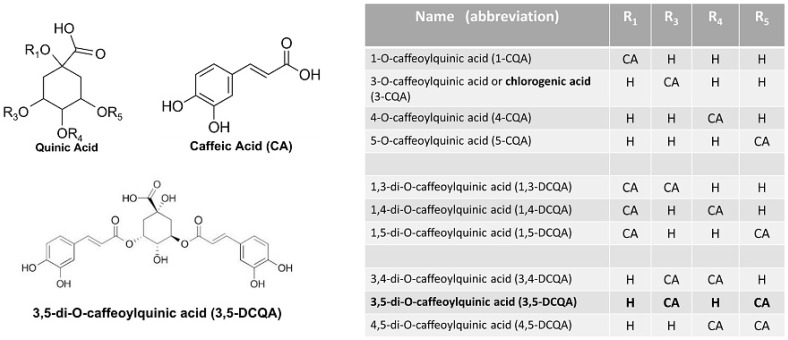
Structure of caffeoylquinic and dicaffeoylquinic acid isomers (IUPAC numbering). CA = caffeic acid.

These natural compounds are esters formed from quinic acid and one or two units of caffeic acid and they are present in plants as multiple isomers (Figure 1). 

In comparison with mono-caffeoylquinic acids, of which chlorogenic acid is the best known, di-caffeoylquinic acid isomers can cross the membranes more rapidly, probably for their greater hydrophobicity [39].

3,5-Dicaffeoylquinic acid (3,5-DCQA or DCQA) is the most abundant di-caffeoylquinic acid isomer present in the *Centella asiatica* extract [40] and in other plant sources [33,34]. In addition, 3,5-DCQA has been reported to inhibit lipids accumulation more potently [41], and it can be absorbed more successfully than other di-caffeoylquinic acid isomers [42], thus increasing bioavailability for both oral and topical applications. 

Although the DCQA bioactivity has been investigated in detail, the molecular mechanisms underlying its anti-adipogenesis action still needs to be clarified. In the present study, we investigated the ability of 3,5-DCQA to inhibit 3T3-L1 adipocyte differentiation. The suppressive effect of DCQA on MCE by increasing Nrf2 and HO-1 expression at an early stage of adipocytes maturation has been attested here. In addition, the DCQA ability to activate AMPK, which in turn lowers FAS expression and ACC activity, suggests the possible use of DCQA as an anti-adipogenic herbal bioactive compound for nutraceutical use.

## 2. Results

### 2.1. DCQA Affects 3T3-L1 Adipogenesis 

3,5-DCQA was chromatoghaphically purified from *Centella asiatica* plant cell cultures in the laboratories of CRODA Italiana S.p.A., Altavilla Vicentina Site, VI, Italy. 

**Figure 2:**
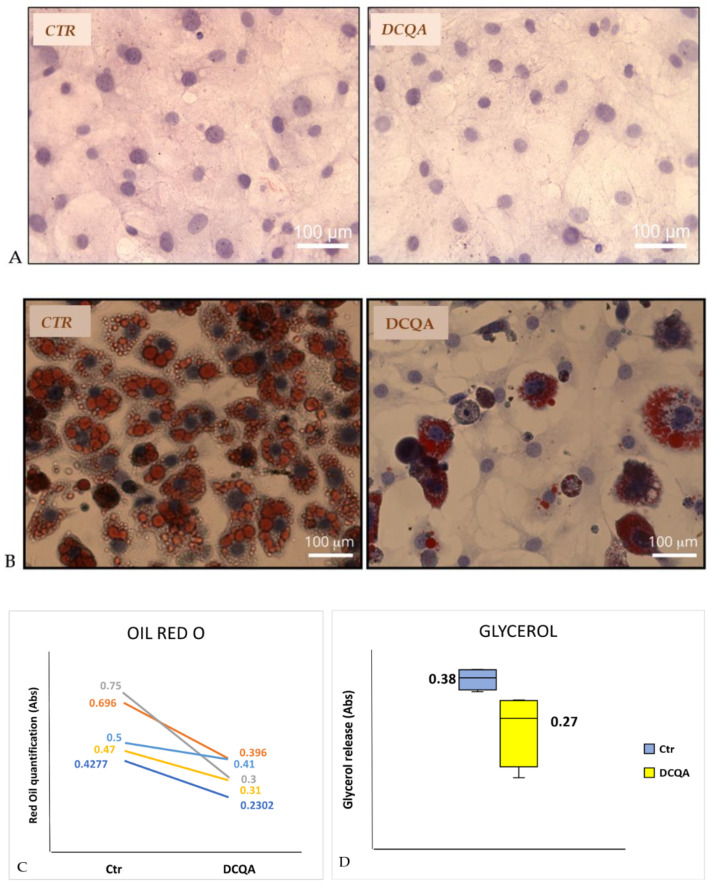
DCQA decreases adipocyte differentiation and glycerol release. (**A**) Pictures represent 3T3-L1 pre-adipocytes after staining with Oil Red O and Hematoxylin. CTR (not-treated sample); DCQA (sample treated with 10 µM DCQA); (**B**) pictures represent terminally differentiated adipocytes after staining with Oil Red O and Hematoxylin. CTR (not-treated sample); DCQA (sample treated with 10 µM DCQA for 10 days); (**C**) quantification of Oil Red O staining at the end of treatment. The absorbance values at 500 nm (for ml of isopropanol) of five independent experiments were shown in the figure. Difference between CTR- and DCQA-treated samples is statistically significant *p* < 0.02 (Student’s *t*-test for paired samples); (**D**) figure shows glycerol release in the culture medium by not-treated cells (CTR) and DCQA-treated cells for all time of differentiation process (DCQA). The difference is statistically significant (*p* < 0.05).

In the absence of any hormonal induction, both treated and not-treated 3T3-L1 pre-adipocytes did not incorporate Oil Red O dye, which stains the triglycerides present in the lipid droplets of cells. In addition, as shown in Figure 2A, 3T3-L1 pre-adipocytes treated with 10 µM DCQA did not changed their fibroblast-like morphology in comparison with the not-treated cells. 

Therefore, the 3T3-L1 adipocytes differentiation process was studied with or without the presence of 10 µM DCQA, added to the culture medium 2 h before MDI stimulation. In the DCQA-treated samples, 10 µM DCQA were added at each change of fresh medium until the end of the treatment. 3T3-L1 cells were induced to differentiate with MDI, as described in the Section 4.2. At the time of terminal differentiation, i.e., at post-induction day (PID) 10, cells were dyed with Oil Red O to determine the degree of differentiation. After the same differentiation period, DCQA-treated 3T3-L1 cells show a significant reduction of Oil Red O staining when compared with non-treated cells (Figure 2B). Based on microscopic observation, all cells of control samples became spherical, accumulated lipid droplets, finally appearing terminally differentiated (Figure 2B, left panel). Instead, many DCQA-treated cells showed no lipid droplets, retaining a pre-adipocyte morphology (Figure 2B, right panel). 

In addition, Oil Red O was solubilized with 1ml isopropanol in each well and quantified at Abs_500_. The mean values ± S.D. of controls vs. DCQA-treated cells were 0.57 ± 0.14 and 0.33 ± 0.07, respectively. Such values were compared by Student’s *t*-test for paired samples reaching the statistical significance (*p* < 0.02). Differences between control and DCQA-treated cells of five independent experiments were shown in Figure 2C.

### 2.2. DCQA Effects on Glycerol Release

To explore whether DCQA can also affect lipolysis, glycerol release in the culture medium was measured. At PID 9, 3T3-L1 adipocytes were cultured for 24 h in a medium without FBS, and after that time, the supernatant was collected. Figure 2D depicts a significant reduction of glycerol release in the culture medium from 10 µM DCQA-treated 3T3-L1 adipocytes in comparison with control cells after normalization to cell number (mean value ± S.D.: CTR sample, 0.38 ± 0.02 vs. DCQA-treated sample, 0.27 ± 0.08; *p* < 0.05). The data suggest that DCQA, which decreased the total amount of lipids stored in the adipocytes, can also reduce lipolysis.

### 2.3. During Terminal Differentiation, DCQA Affects the Expression of Enzymes Controlling Fatty Acid Synthesis

**Figure 3:**
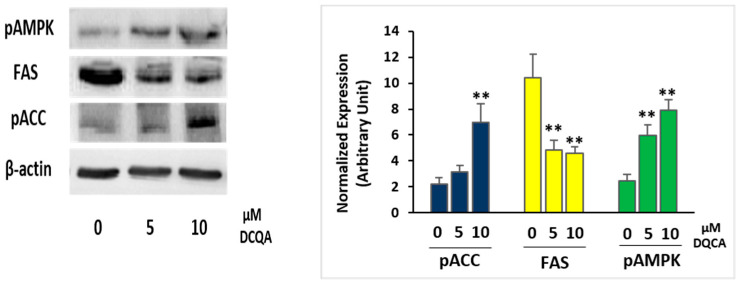
Immunoblot analysis of enzyme involved in the lipid accumulation. 3T3-L1 cells were induced with MDI and treated with or without 5 or 10 µM DCQA for 10 days. At the time of terminal differentiation, cells were collected and immunoblot was performed as described. On the left, a representative experiment is shown. On the right, the quantification of protein expression of three independent experiments is shown. Data from densitometric analyses were normalized with the β-actin expression. Data statistically significant vs. each control sample are indicated. (** *p* < 0.01).

Immunoblot analysis attested that, at time of terminal differentiation (PID10), DCQA-treated 3T3-L1 adipocytes, in comparison with control cells, expressed a significantly higher level of the phosphorylated and active form of AMPK (pAMPK) (Figure 3). Activated AMPK is known to phosphorylate and inactivate acetyl-CoA carboxylase (ACC), which is the rate-limiting enzyme in the fatty acid synthesis pathway [13]. Indeed, an increase in ACC phosphorylation level (pACC), as well as a decreased expression of fatty acids synthase (FAS), has been evidenced (Figure 3).

### 2.4. Effects of DCQA on 3T3-L1 Viability/Cell Proliferation

**Figure 4:**
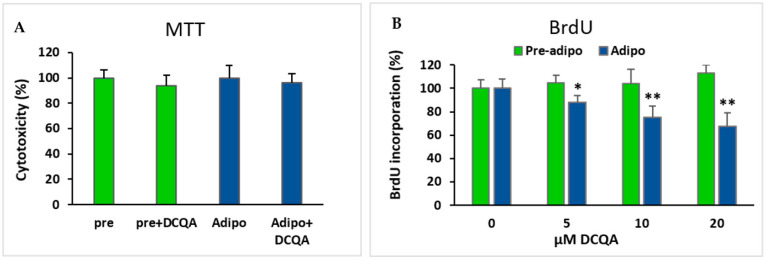
Effect of DCQA treatment on cell viability and cell proliferation (**A**) MTT assay performed after 48 h of treatment with 10 µM DCQA in comparison with control sample. Both pre-adipocytes and 48 h induced adipocytes were compared. Differences are not statistically significant; (**B**) BrdU incorporation assay in not induced (green) and 48 h MDI-induced (blue) 3T3-L1 cells. Cells were treated with 0, 5, 10 or 20 µM DCQA for 48 h. At the end of treatment BrdU incorporation assay did not show any significant difference in pre-adipocytes. On the contrary, in MDI-induced cells for 48 h, a dose-dependent reduction of cell proliferation rate was registered (* *p* < 0.05; ** *p* < 0.001).

To exclude the possibility that the adipogenic decrease could be due to an early toxic effect of DCQA treatment, an MTT viability assay was carried out. Both pre-adipocytes and MDI-induced 3T3-L1 cells were incubated for 48 h with or without 10 µM DCQA. Four hours before the end of the treatment, the MTT reagent was supplemented and maintained in the culture until the treatment was completed. As shown in Figure 4A, the viability of both not-induced and MDI-induced 3T3-L1 cells were not significantly affected by a 48 h DCQA treatment, thus suggesting that no toxic effects were exerted by DCQA during the phase of mitotic clonal expansion.

As the MCE phase is crucial for the subsequent adipocyte terminal differentiation, a BrdU incorporation assay was performed to establish whether DCQA treatment could impair the 3T3-L1 clonal expansion. After culturing cells with 0, 5, 10 and 20 µM DCQA for 48 h, an additional 4 h BrdU incubation showed that 3T3-L1 pre-adipocytes did not significantly reduce the observed proliferation rate at any DCQA concentration tested (Figure 4B, green bars). Conversely, in comparison with not-treated control cells, a significant concentration dependent decrease in BrdU incorporation was found in DCQA-treated 3T3-L1 cells 48 h after MDI induction (Figure 4B, blue bars). This time approximately overlaps to the MCE phase of cell differentiation process. These data suggest that DCQA can lower the rate of proliferation of 3T3-L1 cells elicited to enter in MCE phase by the hormonal cocktail of differentiation. 

### 2.5. In Presence of DCQA, Nrf2 and HO-1 Proteins Were Highly Expressed at the Beginning of the MCE Differentiation Time 

**Figure 5:**
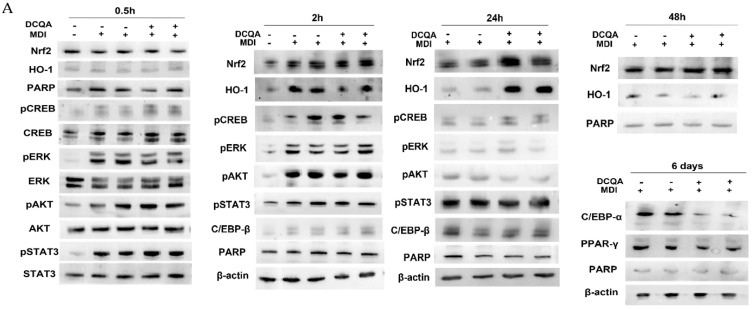

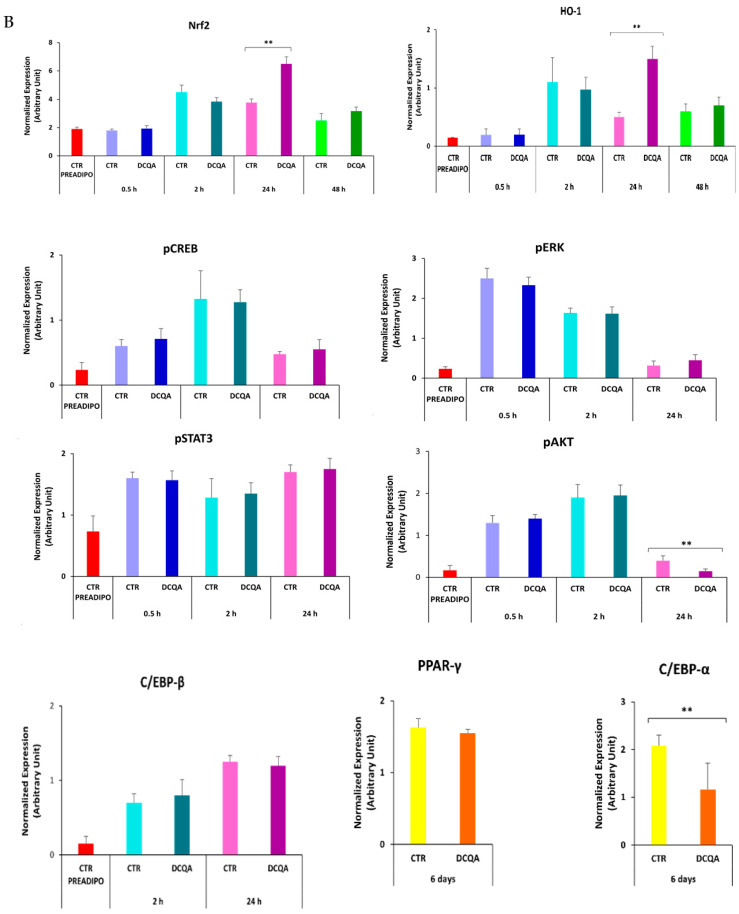
The immunoblots show the expression level of proteins belonging to signaling pathways elicited after MDI stimulation. (**A**) 3T3-L1 cells were induced with MDI for 0.5, 2, 24, 48 h and 6 days, and pre-treated with or without 10 µM DCQA. Immunoblots showed that Nrf2, HO-1, pCREB, pERK and pAkt were activated in comparison with 3T3-L1 cells not stimulated to differentiate. No significative differences are registered between controls and DCQA-treated cells 0.5 and 2 h after MDI stimulation. At 24 h after MDI stimulation, pAkt level decreased, whereas Nrf2 and HO-1 levels increased in DCQA-treated cells vs. controls. At 48 h after MDI stimulation, HO-1 level fell to the level of those registered in the controls at 24 h. At 6 days after MDI stimulation, dissimilarly to PPAR-γ, C/EBP-α expression level decreased in DCQA-treated cells. (**B**) Data from densitometric analyses were normalized with the PARP/β-actin expression. The quantification of protein expression of three independent experiments is shown. Data statistically significant vs. each control sample are indicated. (** *p* < 0.01).

With the purpose of identifying the molecular signals responsible for a decrease in cell proliferation, immunoblot analysis was performed starting from an early time after MDI induction. Ten µM DCQA-treated and not-treated cells were collected at 0.5, 2, 24, 48 h and 6 days after MDI stimulation. At 0.5 and 2 h, the kinases ERK1/2, and Akt, as well as the transcription factors CREB and STAT3, which initiate the earliest phase of adipogenesis, increased their phosphorylated form in comparison with those of quiescent pre-adipocytes (Figure 5). However, they are equally phosphorylated and activated following 0.5 and 2 h’ induction in both DCQA-treated and not-treated cells (Figure 5). Hence, no differences in the activation of the principal early signaling pathways triggered by the hormonal cocktail of differentiation were registered, apparently certifying that DCQA observable effects could occur more than 2 h from induction. 

At 24 h after induction (Figure 5), pERK1/2 returned to the level of not-induced pre-adipocytes, pCREB signal decreased and pSTAT3 and C/EBPβ increased, in comparison with their levels detected earlier, although without any significant differences between DCQA-treated and control samples. However, the most significant variations of pAkt, Nrf2 and HO-1 expression take place at 24 h post-induction. The phosphorylation level of Akt, which was decreased at this time with respect to those detected at 0.5 and 2 h, was further reduced in DCQA-treated cells in comparison with control samples. Moreover, at 24 h post-induction, the expression of both the Nrf2 transcription factor and HO-1 antioxidant protein increased in the DCQA-treated samples when their levels were compared with those of controls at the same time. In actuality, in control samples, the HO-1 expression declined 24 h after MDI stimulation in comparison with its expression at 2 h. Instead, in differentiating conditions, DCQA administration maintains the HO-1 expression at high levels for a further 24 h since HO-1 expression declines only 48 h post induction. Indeed, 48 h after MDI stimulation both the expression levels of Nrf2 and HO-1 were decreased to the level detectable in not-induced pre-adipocytes (Figure 5).

At later phases following MDI induction, the activation of PPAR-γ and C/EBP-α transcription factors are crucial to stop the proliferation phase and stimulate the adipocyte terminal differentiation [7]. At 6 days after induction, PPAR-γ and C/EBP-α were highly expressed in control samples. In DCQA-treated samples, C/EBP-α expression level significantly decreased, while PPAR-γ expression remained unchanged (Figure 5). 

## 3. Discussion

Many experimental and clinical evidences suggest that adipose is a plastic tissue able to be remodeled during pathophysiological conditions. Indeed, despite the fact that terminally developed adipocytes are unable to replicate, progenitors cells resident in adipose tissue can proliferate and then differentiate, supplying new adipocytes to the tissue throughout its life [43,44]. It is also known that the total adipocyte number increases in the subcutaneous depot of woman during overfeeding [45], and that pre-adipocytes from obese subjects proliferate more rapidly in culture than cells from lean individuals [46]. Hence, one way to control obesity, skin lipodystrophy and metabolic disorders associated with energy unbalance is to hinder both hyperplasia, derived by abnormal clonal expansion of progenitor cells, and hypertrophy, i.e., an excessive accumulation of lipid stores that enlarge the size of adipocytes and adipose tissue [45]. 

AMPK activation can regulate carbohydrate and fat metabolism, becoming a potent drug target against obesity and metabolic diseases [13]. In 3T3-L1 pre-adipocytes induced to differentiate, we show that DCQA supplementation, added in the culture medium 2 h before MDI induction, enhances the phosphorylation level of AMPK (Figure 3) at the time of terminal differentiation (PID 10). Accordingly, AMPK target enzymes belonging to the fatty acid synthesis pathway are also affected, as documented by an increase in ACC phosphorylation, resulting in enzyme inactivation and a decrease in FAS protein expression (Figure 3). Consequently, 3T3-L1 cells treated along the differentiation time with 10 µM DCQA display less lipid droplet deposition than not-treated ones, as evidenced by Oil Red O incorporation (Figure 2B,C). Our data agree with a previous report of Karadeniz et al. [47] in which 3T3-L1 adipogenesis, via AMPK activation, was suppressed by the administration of 3,5-dicaffeoyl-epi-quinic acid, an isomer of 3,5-DCQA used by us. Furthermore, other studies converge on the anti-adipogenesis effects of several caffeoylquinic acid compounds purified from different natural sources. A caffeoylquinic acid-rich extract from *Aster glehni* was able to decrease lipid accumulation both in liver and in 3T3-L1 cells [34], and 3,5-DCQA from *Solidago virgaurea* inhibited lipid deposition in the same cell type, although at high concentration [41]. Conversely, Boudreau et al. [48] did not find any effect of 3,5-DCQA on 3T3-L1 cell differentiation, but they measured Oil Red O incorporation after only 4 days from MDI induction, which is before the terminal differentiation takes place. Instead, Duangjai and co-workers [38] reported an inhibitory activity on adipogenesis of a coffee fruit extract containing chlorogenic acid, and other caffeoylquinic acid isomers. In this latter report, the authors additionally showed an increase in lipolysis, although by using an extremely high concentration (200 µM) of quinic acid or chlorogenic acid [38]. On the contrary, we found a reduction of glycerol release from 3T3-L1 cells treated with 10 µM of 3,5-DCQA. We should remark here that the reduction of lipolysis was evident in cell samples treated with DCQA during the differentiation period, at the end of which 3T3-L1 cells became poorly differentiated, still appearing as fibroblast-like pre-adipocytes rather than differentiated adipocytes (Figure 2B). Hence, in such samples, total lipid content was significantly reduced by DCQA-treatment (Figure 2C), justifying less lipolysis assessed by a lower release of glycerol in the culture medium (Figure 2D).

The late AMPK activation could not be the unique target of DCQA anti-adipogenic action. Indeed, the large differences in the maturation state between treated and control cells observed at the time of terminal differentiation suggest that DQCA might affect adipogenesis, starting from an early phase after MDI stimulation. In addition, preliminary results (not shown) indicate a poor reduction of lipid deposition when DCQA treatment began 4 days after MDI induction.

Firstly, we had to rule out the possibility that DCQA treatment could exert an initial cytotoxicity on 3T3-L1 cells. MTT assay carried out 48 h after induction excluded the notion that the DCQA-elicited inhibition of differentiation could be due to an early toxic effect (Figure 4A, blue bars). Accordingly, cell viability of non-hormone-induced pre-adipocytes was not affected by 48 h DCQA treatment as well (Figure 4A, green bars).

As already described, it has been previously demonstrated that cell division blockage during the MCE prevents the subsequent adipogenesis [4,49]. Our experiments of BrdU incorporation carried out 48 h after MDI induction show that DCQA reduces, in a concentration-dependent manner, the proliferation rate of 3T3-L1 cells (Figure 4B, blue bars). Instead, the same treatment did not inhibit the proliferation rate of not-induced pre-adipocytes (Figure 4B, green bars). Hence, we suggest that DCQA pre-treatment can hinder some hyper-proliferative and pro-adipogenic stimuli triggered by MDI administration. The result of this analysis led us to investigate the variations of signaling pathways which occurred in the first days from the MDI stimulation.

Insulin present in MDI can induce ERK1/2 and Akt phosphorylation via Raf/Ras/MEK and IP3K activation, respectively [50]. In Figure 5, immunoblot data show that, in comparison with not-induced pre-adipocytes, the phosphorylated forms of ERK1/2 and Akt were increased early after MDI stimulation. Instead, the immunoblots show no significant change of ERK1/2 and Akt phosphorylation level in DCQA-pre-treated cells collected 0.5 and 2 h after MDI induction, in comparison with the control ones (Figure 5). Only at 24 h the Akt phosphorylation level decreased in the DCQA-treated cells vs. not-treated control, suggesting that the pro-proliferative insulin signaling was turned off at this time in DCQA-treated cells (Figure 5).

Another component of the cocktail of hormones, the IBMX increases cAMP level, the downstream protein kinase A and the transcription factor CREB [6]. The phosphorylation level of CREB was increased at 0.5 and 2 h after adipogenic hormones induction but the DCQA treatment did not inhibit such increase (Figure 5).

The level of STAT3 Tyr705 phosphorylation (pSTAT3) is a sign of its transcriptional activity that supports cell proliferation. Although the pSTAT3 level was high at 0.5, 2 h and at 24 h after MDI stimulation, indicating the pre-adipocytes have been committed to enter in the MCE phase, its phosphorylated level did not change with DCQA treatment as well (Figure 5). 

It was previously reported that the ROS level increased after MDI stimulation [9]. Accordingly, in 3T3-L1 cells, glutathione (GSH) depletion promoted adipogenesis by enhancing both ROS level and proliferation rate; on the contrary, GSH supplementation inhibited adipocytes differentiation [51]. The role of Nrf2 in adipocytes differentiation remains to be clarified, although many Nrf2 activators can inhibit adipogenesis by increasing the intracellular level of GSH and other antioxidants [28,29]. In our experiments, 24 h after MDI stimulation, the Nrf2 protein level increased in DCQA-treated samples. In this regard, chlorogenic acid isomers, including 3,5-DCQA, were reported to protect an intestinal epithelial cell line by the oxidative damage via activated Nrf2 signaling pathway [52]. Furthermore, 5-caffeoylquinic acid ameliorated oxidative stress in hepatocytes by Nrf2 activation [53].

We suggest that, unlike control, the DCQA ability to induce Nrf2 expression could result in maintaining high levels of HO-1 expression. A decline in HO-1 expression, as has been detected in this paper in control cells 24 h after MDI stimulation, is a necessary occurrence for cell cycle progression during the MCE [12]. Therefore, DCQA treatment postponing the decrease in HO-1 expression from 24 h to 48 h results in delaying the advance of MCE phase, likely by maintaining antioxidant conditions for a longer time.

Many other natural compounds can hinder adipogenesis by increasing Nrf2/OH-1 expression and activity [27,28,54,55]. We previously reported that some phenylpropanoid glycosides displayed radical scavenging and antioxidant activities and protected human keratinocytes by inducing Nrf2 and HO-1 expression [56]. It is not surprising that DCQA displayed similar activity on Nrf2 and HO-1 since a caffeic acid moiety is present in both DCQA and phenylpropanoid glycoside structure, thus suggesting a common cytoprotective capability on different cellular systems.

Adipogenesis requires many transcription factors operating in a concerted manner. Following commitment, pCREB induces C/EBPβ expression which firstly promotes pre-adipocytes entering into MCE [57]. We did not find any differences in both pCREB and C/EBPβ expression levels between DCQA-treated and not-treated cells. Nevertheless, the function of C/EBPβ is also regulated by post-translational modifications; after those, it acquires DNA-binding activity transactivating late adipogenic transcription factor C/EBP-α and PPAR-γ genes [7]. C/EBP-α, is actually a pleiotropic activator of the adipogenic process, modulating the expression and activity of lipogenesis effector proteins [7]. We finally found that DCQA-treated 3T3-L1 cells decreased the C/EBP-α expression level 6 days after induction. This completes the molecular mechanism by which DCQA can hinder the sequence of events elicited by MDI stimulation.

## 4. Materials and Methods

### 4.1. Chemicals

3,5-Dicaffeoylquinic acid (DCQA) was provided by CRODA Italiana S.p.A. (Altavilla, Italy). DCQA was purified from crude extracts obtained with a new biotechnological production process based on the use of *Centella asiatica* plant cell culture. For cell treatment, DCQA was solubilized in 100% DMSO at the concentration of 10 mM (1000×). Thereby, the maximal concentration of DMSO in the culture medium was kept low (0.1%). In all tests, the same amount of DMSO (without DCQA) was also added in the culture medium of control samples.

### 4.2. Cell Culture and Differentiation

The mouse embryo-derived 3T3-L1 cell line (ECACC, Merck, Darmstadt, Germany) was used. 3T3-L1 cell line displays a fibroblast-like, pre-adipocyte signature, but can undergo adipocyte conversion with appropriate stimuli. The 3T3-L1 cells were cultured in high glucose Dulbecco’s Eagle Medium (DMEM, Gibco, Thermo Fisher Scientific, Waltham, MA, USA) supplemented with 10% fetal bovine serum (FBS, Gibco, Thermo Fisher Scientific, Waltham, MA, USA) and 1% Antibiotic-Antimycotic solution (Gibco, Thermo Fisher Scientific, Waltham, MA, USA) at 37 °C in a humidified atmosphere of 5% CO_2_. Once 90% of confluency was reached, 3T3-L1 cells were detached using trypsin-EDTA (Gibco, Thermo Fisher Scientific, Waltham, MA, USA) and seeded. Two days after confluency, 3T3-L1 differentiation process was induced by replacing DMEM with the differentiation medium (MDI), consisting of DMEM/F12 (Gibco, Thermo Fisher Scientific, Waltham, MA, USA), supplemented with 10% FBS, 1% Antibiotic-Antimycotic solution, 0.2 mM IBMX (Merck, Darmstadt, Germany), 10 µM Rosiglitazone (Rosi, Merck, Darmstadt, Germany), 1 µM Dexamethasone (Dex, Merck, Darmstadt, Germany) and 10µg/mL insulin (Merck, Darmstadt, Germany). Three days later, at post-induction day (PID)-3, cells were placed in maintenance medium, composed of DMEM/F12 enriched with 10% FBS, 1% Antibiotic-Antimycotic solution and 10 µg/mL insulin. Two days later, (PID-5), the medium was replaced with DMEM/F12 enriched with 10% FBS, and 1% Antibiotic-Antimycotic solution. Thereafter, the fresh medium was replaced every 2 days. In the DCQA-treated plates, 10 µM DCQA was added at each change of fresh medium until the end of the treatment. 3T3-L1 cells differentiated to mature adipocytes in about 10 days (PID-10). 

### 4.3. Cell Treatment

3T3-L1 pre-adipocytes were treated with DCQA 2 h (h) before MDI stimulation and until the mature adipocytes differentiation. Cell samples were harvested before (pre-adipocytes) and 0.5 or 2 h after MDI induction for the immediate early protein expression, as well as 24, 48 h and 6 days after MDI induction, and at the terminal differentiation time (10 days) for immunoblot analysis. Cells at different time points were collected for other assays, as the following described. When not otherwise specified, “control samples” refers to 3T3-L1 cells induced to differentiate with MDI but not treated with DCQA.

### 4.4. Oil Red O Staining

Pre-adipocytes were seeded (10^5^ cells /well) in a 6-well plate containing pre-sterilized coverslips. Before inducing differentiation, cells were treated with/without DCQA at the final concentration of 10 µM until terminal differentiation. After maturation, adipocytes were washed with phosphate saline buffer (PBS, Gibco, Thermo Fisher Scientific, Waltham, MA, USA), fixed with 10% formalin for 10 min and washed twice with deionized water for 5 min. Cells were stained with Oil Red O solution (Merck, Darmstadt, Germany) for 10 min, and then washed three times with deionized water. Nuclei were stained with Hematoxylin (Bio-Optica, Bologna, Italy). The stained lipid droplets were visualized using an inverted microscope, EVOS FL Auto Cell Imaging System (Thermo Fisher Scientific, Waltham, MA, USA). In separate samples, Oil Red O dye was eluted with 1 mL isopropanol per well and the Abs_500_ nm was quantified using Tecan NanoQuant Infinite M200 Pro reader (Tecan Group Ltd., Männedorf, Switzerland).

### 4.5. Glycerol Release Assay

To perform the glycerol release assay, at the terminal differentiation time (PID 9), cells were incubated in culture medium without FBS for 24 h. After that, the supernatant was collected, and glycerol content measured according to manufacturer’s instructions (Free Glycerol Assay Kit; ab65337, Abcam, Cambridge, UK). Glycerol content was normalized to cell viability measure using crystal violet staining solution (Merck, Darmstadt, Germany) as previously described [58].

### 4.6. Cell Viability Assay

3T3-L1 cells were seeded in 96-well plates at a density of 2 × 10^3^ cells/well. Cells were treated for 48 h with a DMSO solution at the final concentration of 10 µM DCQA in the presence or absence of a cocktail of differentiation (MDI). Cell viability was evaluated by 3-(4,5-dimethylthiazol-2-yl)-2,3-diphenyl tetrazolium bromide (MTT) assay. Briefly, 4 h before the end of the treatment, MTT reagent (0.5 mg/mL, 10 µL) was added to each well and incubated until treatment was completed. The colored crystals of produced formazan were dissolved in 200 µL of isopropanol and the Abs_500_ was measured in the plate reader Tecan NanoQuant Infinite M200 Pro (Tecan Group Ltd., Männedorf, Switzerland). Four replicates for each condition were performed. Three independent experiments were performed for each condition.

### 4.7. 5-Br-2′-Deoxy-Uridine Cell Proliferation Assay

Cell proliferation was assessed with a colorimetric immunoassay (BrdU Elisa Kit; Merck, Darmstadt, Germany) based on the measurement of 5-Br-2′-deoxy-Uridine (BrdU) incorporation into DNA of the cells during the S phase. In 96-well plates, 0, 5, 10 and 20 µM DCQA were added to the culture medium of 3T3-L1 pre-adipocytes. DCQA compound remained present in the culture medium during all time of the subsequent induction. Two hours later, 3T3-L1 cells were or not induced to differentiate with MDI for the indicated times. Four hours before the end of induction time, BrdU was added to each well. At the end of treatment, the medium was aspirated, and cells were incubated with a fixing-denaturing solution for 30 min. Plates were washed three times and the anti-BrdU detector antibody was added for 1 h and a half. Plates were incubated for 30 min at RT with peroxidase goat anti-mouse IgG conjugate and then washed three times. The tetramethylbenzidine chromogenic peroxidase substrate was added, and plates were incubated for 5 min in the dark. Finally, a stop solution (1M H_2_SO_4_) was added and the Abs_450_ measured using the Tecan NanoQuant Infinite M200 Pro reader (Tecan Group Ltd., Männedorf, Switzerland).

### 4.8. Immunoblot Analysis

Total cell lysate was prepared using 2 mM EDTA pH 8, 20 mM Tris HCl pH 7.5, 1% Triton X-100, 0.1% SDS buffer. Lysis was performed at 4 °C for 40 min. Cell lysate was centrifuged at full speed for 20 min. Pellet was discarded and proteins concentration in the supernatant were determined by Bradford assay, measuring Abs_495_ in a spectrophotometer Jasco V-650 (Jasco Europe, Cremella, Italy). A total of 30 µg of each sample was added with 5× sample buffer (50mM Tris HCl pH 6.8, 2% SDS, 10% glycerol, 5% β-mercaptoethanol, 0.1% bromophenol blue) and heated for 5 min at 95 °C. Samples were loaded on 7.5 or 12% SDS-polyacrylamide gel electrophoresis. After electrophoresis sample were transferred to polyvinylidene difluoride membrane (PVDF, Thermo Fisher Scientific, Waltham, MA, USA) and then blocked for 1 h with TBS-T (10 mM Tris HCl pH 7.5, 100 mM NaCl, 0.1% Tween 20) containing 5% bovine albumin serum (BSA, Merck, Darmstadt, Germany) or 4% milk. Membranes were incubated overnight with a solution of 5% BSA containing a primary antibody: pAMPKα (Thr172), pAkt (Ser473), pSTAT3 (Tyr705), pERK1/2 (Thr202/Tyr204), Akt, C/EBP-β and β-actin, 1:2000 (Cell Signaling Technology, Danvers, MA, USA); pACC (Ser79), FAS, pCREB (Ser133), PARP, Nrf2, HO-1, STAT3, CREB, ERK1/2, 1:3000 (Genetex, Alton Pkwj Irvine, CA, USA); C/EBP-α, PPAR-γ, 1:1000 (Santa Cruz Biotechnology, Dallas, TX, USA). Thus, membranes were washed three times with TBS-T buffer for 30 min and incubated with horseradish peroxidase-conjugated secondary antibody (anti rabbit 1:4000/1:6000 (Cell Signaling Technology, Danvers, MA, USA) in TBS-T buffer containing BSA 5% for 1 h. Before blot processing with ECL solution (Thermo Fisher Scientific, Waltham, MA, USA) and chemiluminescence signal visualization on ChemiDoc Universal Hood II (Bio-Rad Laboratories Srl, Segrate, Mi, Italy), the membranes were washed again 3 times for 30 min.

### 4.9. Statistics

All the results are reported as a mean value ± SD. Unless noted otherwise, p values were determined using an unpaired, two-tailed Student’s *t*-test. For each type of experiment, a minimum of three independent biological replicates were performed.

## 5. Conclusions

3,5-DCQA and its isomers are commonly present in many herbal extracts and abundant phenolic compounds in coffee as well. Indeed, pharmacokinetic data identified the presence of 3,5-DCQA in plasma of healthy subjects after one coffee consumption, with a C_max_ of about 1 µM [59]. That proves the bioavailability of this compound supporting its use as nutraceutical. 

Our study attested that 10 µM DCQA treatment can hinder 3T3-L1 adipogenesis, leading to a significative reduction of lipid deposition at the end of differentiation process. At this time, an AMPK activation occurred, also inhibiting fatty acid anabolism. Furthermore, we identified other important DCQA-affected processes which begun earlier after MDI stimulation. At about 24 h post-induction, the expression level of the transcription factor Nrf2 and the antioxidant protein HO-1 were increased in comparison with controls. Because a decrease in HO-1 expression and a consequent increase in ROS level are mandatory for 3T3-L1 cells entry the mitotic clonal expansion, the high HO-1 expression resulted in a reduction of cell proliferation rate, as registered by a BrdU assay. This effect results in lower expression of C/EBP-α and lower number of cells able to mature in adipocytes. This molecular mechanism, which might be common to other compounds containing caffeic acids moiety, could be exploited to reduce obesity onset and progression by hindering both hyperplasia and hypertrophy of adipose tissue. It must be highlighted that an overexpression of HO-1 has been demonstrated to also play a beneficial role in metabolic diseases linked to obesity [60].

## Data Availability

Not applicable.

## Sample Availability

Not available.
